# Increased autophagy signaling but not proteasome activity in human skeletal muscle after prolonged low‐intensity exercise with negative energy balance

**DOI:** 10.14814/phy2.13518

**Published:** 2017-12-06

**Authors:** Marcus Moberg, Gina Hendo, Madelene Jakobsson, C. Mikael Mattsson, Elin Ekblom‐Bak, Mikael Flockhart, Marjan Pontén, Karin Söderlund, Björn Ekblom

**Affiliations:** ^1^ Åstrand Laboratory of Work Physiology the Swedish School of Sport and Health Sciences Stockholm Sweden

**Keywords:** Energy deficit, LC3b, military training, mTORC1, proteolysis, ULK1

## Abstract

Little is known about the molecular regulation of skeletal muscle protein turnover during exercise in field conditions where energy is intake inadequate. Here, 17 male and 7 female soldiers performed an 8 days long field‐based military operation. Vastus lateralis muscle biopsies, in which autophagy, the ubiquitin–proteasome system, and the mTORC1 signaling pathway were studied, were collected before and after the operation. The 187 h long operation resulted in a 15% and 29% negative energy balance as well as a 4.1% and 4.6% loss of body mass in women and men, respectively. After the operation protein levels of ULK1 as well as the phosphorylation of ULK1^Ser317^ and ULK1^Ser555^ had increased by 11%, 39%, and 13%, respectively, and this was supported by a 17% increased phosphorylation of AMPK^Thr172^ (*P *<* *0.05). The LC3b‐I/II ratio was threefold higher after compared to before the operation (*P *<* *0.05), whereas protein levels of p62/SQSTM1 were unchanged. The *β*1, *β*2, and *β*5 activity of the proteasome and protein levels of MAFbx did not change, whereas levels of MuRF‐1 were slightly reduced (6%, *P *<* *0.05). Protein levels and phosphorylation status of key components in the mTORC1 signaling pathway remained at basal levels after the operation. Muscle levels of glycogen decreased from 269 ± 12 to 181 ± 9 mmol·kg dry·muscle^−1^ after the exercise period (*P *<* *0.05). In conclusion, the 8 days of field‐based exercise resulted in induction of autophagy without any increase in proteasome activity or protein ubiquitination. Simultaneously, the regulation of protein synthesis through the mTORC1 signaling pathway was maintained.

## Introduction

Exercise and nutritional intake regulate skeletal muscle protein turnover by altering the rates of muscle protein synthesis and/or muscle protein breakdown. The rate of protein synthesis is under control by the mechanistic target of rapamycin complex 1 (mTORC1) pathway, which is potently stimulated by contractile activity and ingestion of amino acids (Drummond et al. 2009b; Dickinson et al. 2011). In contrast, reduced contractile activity, energy deficit, and amino acid insufficiency downregulates mTORC1 signaling and the rate of protein synthesis (Ferrando et al. 1997; Hara et al. 1998; Pasiakos et al. 2010; Drummond et al. 2012; Vendelbo et al. 2014). The effects of training, laboratory‐based exercise, and various aspects of nutritional intake on mTORC1 signaling are extensively studied in skeletal muscle (Drummond et al. 2009a; Goodman 2014; Marcotte et al. 2015). However, there is a shortage of studies examining how the mTORC1 signaling pathway responds under conditions such as adventure racing or military operations, where low‐ and high‐intensity exercise is combined and nutritional intake often is inadequate.

The rate of protein breakdown is increased after exercise in the postabsorptive state (Phillips et al. 1997) as well as after a period of energy deficit (Carbone et al. 2014) to remove damaged proteins, enable remodeling, and provide amino acid substrates. In comparison to the cellular control of protein synthesis the regulation of protein breakdown is more versatile and primarily revolves around two systems, the autophagy–lysosomal and the ubiquitin–proteasome system (Sandri 2013). For the latter, the degradation process starts with protein ubiquitination which is controlled by ubiquitin ligases (E3s) (Reid 2005) such as Muscle atrophy F box (MAFbx) and muscle ring finger‐1 (MuRF‐1) (Bodine et al. 2001; Gomes et al. 2001) which are the two most extensively studied E3s in skeletal muscle. Their critical role in proteolytic regulation have been illustrated in various genetic knock out models, as well as by the upregulated expression found in numerous atrophy models in human muscle (Bodine and Baehr 2014). Conversely, several studies have also found that both endurance and resistance exercise increases the expression of particularly MuRF‐1 in human muscle (Yang et al. 2006; Louis et al. 2007; Mascher et al. 2008; Apro et al. 2015), suggestively to support muscle tissue remodeling and exercise adaptations. It is, however, not well described how their expression is altered when exercise is severely prolonged and a catabolic state is manifested.

Studies of the influence of exercise and nutrition on proteasome activity in human muscle are spares and results are disperse. Jamart and colleagues (Jamart et al. 2012) found increased *β*2‐proteasome activity following a 24 h ultramarathon with concomitant energy deficit, whereas *β*1 and *β*5 activity was unaltered. In contrast, reduced *β*5 activity has been noted after a 200 km endurance run (Kim et al. 2011a), whereas 45 min moderate intensity running had no impact on the corresponding activity (Carbone et al. 2014). Although energy deficit per se has not been shown to induce proteasome activity, provision of amino acids by protein ingestion reduces the activity following exercise or after a period of energy deficit (Lunn et al. 2012; Carbone et al. 2013).

Initiation of the bulk degradation process of macroautophagy (herein autophagy), is controlled by the unc‐51 like autophagy activating kinase 1 (ULK1) protein complex which is inhibited through phosphorylation by mTORC1 under nutrient rich conditions and activated by AMP‐activated protein kinase (AMPK) during, for example energy stress (Parzych and Klionsky 2014). Expansion and final formation of the autophagosome involves the lipidation of microtubule‐associated protein1 light chain 3 beta (LC3b‐I), forming LC3b‐II which attaches to the autophagosome membrane (Parzych and Klionsky 2014). In autophagosome formation p62/sequestosome‐1 (p62/SQSTM1) binds to both aggregated proteins and LC3b‐II and ultimately gets degraded in the autolysosome. Autophagy is shown to be required for the maintenance of muscle mass (Masiero et al. 2009) and exercise adaptations in rodents (He et al. 2012; Lira et al. 2013). In human muscle, exercise stimulated autophagy signaling and autophagosome formation was first shown following ultraendurance running (Jamart et al. 2012), and subsequently illustrated during shorter and more applicable exercise durations (Moller et al. 2015; Fritzen et al. 2016). Nutrient provision or insulin stimulation reduces autophagy in human muscle (Vendelbo et al. 2014; Fritzen et al. 2016), whereas fasting induces autophagosome formation although the effect seems to be less potent than exercise‐induced stimulation (Sanchez et al. 2014; Vendelbo et al. 2014).

Our understanding of the influence of exercise and nutrition on the regulation of autophagy in human skeletal muscle is, however, in its infancy and a better overall picture of the regulation of proteolysis need to be formed. Accordingly, the objective of this study was to examine the effect of a prolonged period with low‐ and high‐intensity weight bearing exercise, combined with a negative energy balance, on proteasome activity, autophagy, and mTORC1 signaling in human skeletal muscle. The aim was to increase our knowledge of the molecular regulation of protein turnover during physically strenuous efforts such as military operations, adventure racing or expeditions that are almost inherently catabolic. For this purpose 17 male and 7 female soldiers were studied before and after an 8 day (187 h) field‐based military training operation with inadequate nutritional intake. We hypothesized that the exercise period would have a negative impact on protein levels and signaling in the mTORC1 pathway as well as induce proteasome activity and autophagy.

## Material and Methods

Twenty‐four healthy soldiers from a larger troop, 7 women, 22 ± 1 (19–34) years, 70.9 ± 2.5 (59.7–79.8) kg, and 17 men, 20 ± 1 (19–23) years, 80.3 ± 1.6 (71.3–92.6) kg were recruited for a military training operation. The operation lasted for 8‐days (187 h) consisting of mixed types of physical activity in varying terrain and while moving soldiers carried gear with a total weight of 35 kg. After information of the purpose of the study and of associated risks, all the 24 subjects gave their written consent. The study was approved by the Regional Ethical Review Board in Stockholm and performed in accordance with the principles outlined in the Declaration of Helsinki.

Before the military exercise, and more than three hours after the last meal, different measurements and tests were conducted. First, at rest a 5 mL venous blood sample was obtained for analyses of different blood parameters. Secondly, body weight was measured with standardized methods within the nearest 0.1 kg. Subsequently, a counter movement jump with free arms (CMJa), a squat jump (SJ), both proxies for dynamic leg muscle strength, and in addition an isometric hand grip test (HG) for both hands were carried out using common test prerequisites. For these measurements the best result of two attempts were recorded. Next, for estimation of VO_2max_ a submaximal bicycle test using the Ekblom‐Bak methodology was carried out (Ekblom‐Bak et al. 2014). Finally, resting muscle biopsy specimens (two pieces) was obtained under local anesthesia using 2–3 mL Xylocain (Lidocaine, AstraZeneca AB, Södertälje, Sweden) from the distal portion of *Vastus Lateralis* of the *Quadriceps Femoris* muscle of one leg using a Weil–Blakesley conchotome (AB Wisex, Mölndal, Sweden) under standardized medical supervision (Ekblom 2017).

During the 187 h exercise the participants carried a heart rate monitor (chest band and watch, RS800, Polar Electro Oy, Finland). The average heart rate during the whole operation in combination with estimated *V*O_2max_ using the individual relationship between heart rate and standard values of oxygen uptake from the two work rates during the submaximal cycle exercise was used for an estimation of energy expenditure. There was no correction in estimated energy expenditure with regard to variations in RQ. In this calculation a RQ value of 0.85 was used.

Energy intake was evaluated from the amount of standard field food rations given to each group of soldiers. The food was freeze‐dried and consisted in average of 14% protein, 35% fat, and 51% carbohydrates. In between meals even some minor snacks (including a combination of protein, fat, and carbohydrates) were ingested. It should be emphasized that total amount of food actually ingested, as well as the exact content of macronutrients, are difficult to calculate during field conditions, since not all food was ingested for different reasons, and furthermore food is not uncommonly exchanged between participants. All things considered, the figures for both energy expenditure and energy intake are only rough group estimates and cannot be used for individual evaluations of energy expenditure, energy intake or energy deficit. After eight days (187 h) of military exercise the operation was terminated and soldiers returned to the military facilities to immediately complete the series of physiological tests. The order of the tests were the same as before the operation and with the final meal of the operation ingested at least three hours prior to termination. The skeletal muscle biopsies were collected 60–120 min after cessation of exercise. Exact biopsy timing varied between subjects since the soldiers arrived in groups of eight to complete the tests, all, however, collected within that 1–2 h post operation period. After collection all the biopsies were quickly blotted free from visible blood, fat, and connective tissue and rapidly frozen in liquid nitrogen and subsequently stored at −80°C until further analysis. Unfortunately, due to issues during sampling and low quality some of the sampled biopsies, muscle tissue from Pre or Post time points was not sufficient for all designated analysis in up to 12 subjects.

### Blood sample analysis

Following blood samples collection in heparinized tubes samples were kept on ice prior to centrifugation at 3000*g* for 10 min. The plasma thus obtained were transferred to new tubes, immediately frozen in liquid nitrogen and stored at −80°C until further analysis. Plasma samples were analyzed for levels of free fatty acids (FFA), cholesterol, IL‐6, cortisol, testosterone, and IGF‐1. All the analyses were carried out at the Laboratory of Clinical Chemistry at the Karolinska Hospital using standardized and validated clinical laboratory methods.

### Muscle glycogen

One part of the muscle biopsy was used for glycogen determination using a modified method according to the method described by Harris et al. (1974). Briefly 0.5–2.5 mg of freeze‐dried and dissected muscle was digested in 0.1 mol·L^−1^ NaOH at 80° for 10 min and then neutralized with 0.1 mol·L^−1^ HCl + 0.2 mol·L^−1^ citric acid + 0.2 mol·L^−1^ Na_2_HPO_4._ Amyloglucosidase was added to hydrolyze glycogen to glucose. The amount of glucose was determined spectrophotometrically using an enzymatic method modified from Bergmeyer (1974).

Muscle samples from three subjects (one male and two female participants) could not be analyzed for muscle glycogen concentration after the exercise due to technical difficulties. Therefore in this part of the study muscle specimens from 21 participants before and after the exercise were analyzed.

### Citrate synthase and HAD activity

The activity of citrate synthase and *β*‐hydroxyacyl‐Coenzyme A dehydrogenase was measured using freeze‐dried and dissected muscle tissue according to Psilander et al. (2015).

### Immunoblotting

The immunoblotting process was performed, with minor modifications, as described in detail in the paper by Moberg et al. (2016). In brief, 3 mg muscle samples from 13 subjects (4 women and 9 men; due to insufficient amount of muscle from the remaining 11 subjects) that had been freeze‐dried and dissected clean from blood and connective tissue were homogenized in ice‐cold buffer (100 *μ*L·mg·dry·muscle^−1^) consisting of 40 mmol·L^−1^ HEPES, 120 mmol·L^−1^ NaCl, 10 mmol·L^−1^ NaPPO_4_, 50 mmol·L^−1^ NaF, 1 mmol·L^−1^ EDTA, 0.3% CHAPS, 1% (v/v) phosphatase inhibitor cocktail (Sigma P‐2850), and 1% (v/v) Halt Protease Inhibitor Cocktail (Thermo Scientific, Rockford) using a BulletBlender (NextAdvance) and 0.5 mmol·L^−1^ ZrO beads. Samples were subsequently centrifuged at 10 000*g* for 10 min at 4°C and the supernatant was collected and determined for protein concentration using Pierce^TM^ 660 nm protein assay (Thermo Scientific). Protein separation (30 *μ*g) was performed using precast Criterion TGX gradient gels (4–20% acrylamide, Bio‐Rad Laboratories) and electrophoresis was conducted on ice for 30 min at 300 V in a cold room. Gels were subsequently equilibrated in transfer buffer (25 mmol·L^−1^ Tris base, 192 mmol·L^−1^ glycine, and 10% methanol) for 30 min and then transferred to polyvinylidine fluoride membranes (Bio‐Rad Laboratories) on ice for 180 min at 300 mA.

To confirm equal loading and transfer, the membranes were stained with MemCode^TM^ Reversible Protein Stain Kit (Thermo Scientific). Following destaining, membranes were blocked for 60 min in Tris‐buffered saline (TBS) supplemented with 5% nonfat dry milk and thereafter incubated overnight with commercially available primary antibodies diluted in TBS supplemented with 0.1% Tween and 2.5% nonfat dry milk (TBS‐TM). Membranes were then washed serially with TBS‐TM and incubated for 60 min with horseradish peroxidise conjugated secondary antibodies at room temperature. After serial washes with TBS‐TM and TBS the membranes with antibodies bound to each target protein were visualized by applying chemiluminescent substrate (Super Signal^TM^ West Femto, Thermo Scientific) to the membranes, followed by detection and on a Molecular Imager ChemiDoc^TM^ XRS system and subsequent quantification of the resulting bands.

Prior to blocking, membranes from each gel were cut in stripes for each target protein and then assembled. Thus, all membranes with samples from each subject were exposed to the same blotting conditions. All values obtained for total protein abundance were related to the total protein stain at ~95 kDa obtained with the Memcode kit, which has a coefficient of variation in 2.4% in our laboratory. Levels of each phosphorylated protein were expressed in relation to corresponding level of total protein. Following visualization, in the case of 4E‐BP1, eEF2, and AMPK, the membranes were stripped of the phosphospecific antibodies after which the membranes were washed and reprobed with primary antibodies for each respective total protein as described above.

Primary antibodies for total mTOR, total S6K1, total 4E‐BP1, eEF2^T56^, total eEF2, AMPK^T172^, total AMPK, ULK1^S318^, ULK1^S555^, ULK1^S638^, Ubiquitin, Beclin1, p62/SQSTM1, LC3b and the secondary rabbit antibody came from Cell Signaling Technology (Beverly), primary 4E‐BP1^T46^, MuRF‐1 from Santa Cruz Biotechnology (Santa Cruz) and primary total ULK1, MAFbx as well as the secondary goat antibody from Abcam (Cambridge, England).

### Proteasome activity

The activity of the chymotrypsin‐like‐(*β*5), trypsin‐like‐(*β*2), and caspase‐like‐(*β*1) subunits of the proteasome was measured using the commercially available Proteasome‐Glo^TM^ Assay Systems kit (Promega Biotech, Nacka, Sweden). Two mg of lyophilized muscle tissue from 12 subjects (4 women and 8 men; due to insufficient amount of muscle from the remaining 12 subjects) was homogenized in 300 *μ*l ice‐cold buffer (50 mmol·L^−1^ Tris, 150 mmol·L^−1^ NaCl, 5 mmol·L^−1^ MgCl_2_, 1 mmol·L^−1^ EDTA, 1 mmol·L^−1^ DTT; pH 7.5), using a BulletBlender (NextAdvance) and 0.5 mmol·L^−1^ ZrO beads, for 2 min at 4°C. Homogenates were subsequently rotated for 30 min at 4°C and centrifuged for 10 min at 10 000*g,* after which the protein concentration was determined in the collected supernatant (average 1.65 *μ*g·*μ*L^−1^).

The supernatant was subsequently diluted with 10 mmol·L^−1^ HEPES (pH 7.6) in two aliquots with 0.4 *μ*g protein·*μ*L^−1^, one containing 1 mmol·L^−1^ of the proteasome inhibitor MG‐132, thus serving as a negative control, which was incubated with inhibitor for 60 min on a rocking platform in a cold room immediately prior to the activity assays. The assays were performed in duplicate on 96‐well, white wall, plates with 10 *μ*g of protein from each sample or negative control in each reaction. The assays were initiated by the addition of kit specific assay buffer containing either Suc‐LLVY (*β*5), Z‐LRR (*β*2) or Z‐nLPnLD (*β*1) substrate, and then incubated for 30 min in room temperature before luminescence (relative light units; RLU) was recorded on a GloMax^TM^ luminometer (Promega Biotech). For each sample, plus negative control, the activity of all three proteasome subunits was measured on the same plate.

One third aliquot of the initial homogenate was, prior to the assays, diluted in Laemmli sample buffer to a final concentration of 0.5 *μ*g·*μ*L^−1^, heated to 95°C for 5 min, and subjected for immunoblotting to determine proteasome protein content. Western blotting was conducted as described above, but with 10 *μ*g of protein for separation, and blotted for the 19S regulatory S4 subunit as well as the 20S core subunits (Enzo Life Sciences, Farmingdale, NY #PW‐9355 and #PW‐0530).

The RLU from each sample (mean of duplicate) was normalized against the protein content of both the 19S S4 subunit and the 20S core subunits after subtracting the RLU from corresponding negative control. Prior to conducting the assays for the study, methodological validation was performed using purified 26S proteasome and human skeletal muscle collected at rest and after high‐intensity exercise in the fasted state. The standard curves for *β*5, *β*2, and *β*1 using purified 26S proteasome (0–500 ng) all had a coefficient of variation (*r* ^2^) above 0.99. Performing standard curves using human skeletal muscle protein (0 to 25 *μ*g) the coefficients of variation were 0.99 (*β*5), 0.94 (*β*2), and 0.98 (*β*1). The blocking of proteasome activity using MG‐132 was 99.5%, 96.3%, and 99.3% complete for chymotrypsin‐like, trypsin‐like, and caspase‐like, respectively, after subtracting for a blank sample (no protein). Using the standard curves, the average activity at rest, for the samples in the study, corresponded to the activity of 23 ng purified 26S proteasome for *β*5, 47 ng for *β*2, and 78 ng for *β*1.

### Statistical analysis

Parametric statistical procedures were employed to calculate the means and standard error (SEM), which accordingly are the values presented. For comparison between Pre to Post changes the Students′ paired *t*‐test was used, whereas an unpaired *t*‐test was employed to evaluate differences between males and females. A *P*‐value of *P *<* *0.05 was considered statistical significant.

## Results

### Body weight

Body weight for women was 70.9 ± 2.5 kg before and 68.0 ± 2.4 kg after the exercise period (*P *<* *0.05). For men the corresponding values were 80.3 ± 1.6 kg and 76.6 ± 1.5 kg (*P *<* *0.05). Accordingly women had an average weight reduction in 4.1% and men 4.6%.

### Energy expenditure and intake

Heart rate including sleep and other rest periods in the participants, in whom recordings could be evaluated for the entire exercise, averaged at 88 beats per min. However, during about a total of 2.5 h, divided over nine occasions, heart rate was at 140–190 beats per min, indicating intense bouts of exercise interspersed in the low‐intensity exercise. During the entire operation the soldiers spent 16–19% of the time at rest. The mean value of energy expenditure during the whole operation, including rest periods, was estimated to 39,000 kcal for women and 46,500 kcal for men, corresponding to 4900 and 5800 kcal per 24 h, respectively. When accounting for body weight the energy expenditure varied from 2.8 to 3.4 kcal·kg^−1^·h^−1^. The mean value of estimated energy intake was 33,000 kcal for both women and men, leading to an overall total estimated energy deficit of about 6000 kcal (15%) and 13,500 kcal (29%) for women and men, respectively.

### Muscle strength tests

The height of the two jump test was significantly reduced after compared to before the exercise period for the whole group, with no difference in the reduction between women and men. For CMJa jump height was 37.8 ± 1.6 cm before and 35.6 ± 1.6 cm after the exercise (*P *<* *0.05 vs. Pre). Corresponding values for the SJ were 26.9 ± 1.1 cm before and 25.7 ± 1.2 cm (*P *<* *0.05 vs. Pre). For the dominant hand the grip strength test values before compared to after the operation was significantly reduced, values being 523 ± 22 kp and 498 ± 23 kp, respectively, and for the nondominant hand 479 ± 22 kp and 463 ± 23 kp, respectively (all *P *<* *0.05 vs. Pre).

### Oxygen uptake

The estimated *V*O_2max_ for women was 3.42 ± 0.13 L·min^−1^ before and 3.44 ± 0.16 L·min^−1^ after the exercise, and for men 4.35 ± 0.04 and 4.17 ± 0.05 ·min^−1^, respectively, which was a significant reduction from before to after the military exercise for the males (*P *<* *0.05). Corresponding values per kg body weight were 47.2 ± 2.0 and 48.9 ± 2.3 mL·min^−1^·kg·bw^−1^ for women and 54.3 ± 0.04 and 54.6 ± 0.08 mL·min^−1^·kg·bw^−1^ for men (nonsignificant changes).

### Plasma values

Free fatty acids (FFA) increased from 0.09 ± 0.01 to 0.15 ± 0.04 mmol·L^−1^ and cholesterol decreased from 4.4 ± 0.2 to 4.0 ± 0.1 mmol·L^−1^ after the exercise period (*P *<* *0.05), without differences between women and men. Preexercise levels of cortisol varied greatly between participants (range 66 to 463 nmol·L^−1^), with the mean value being unchanged after compared to before the operation, with numerically higher levels in men (183 ± 31 nmol·L^−1^) than in women (131 ± 17 nmol·L^−1^). Total testosterone concentration decreased in the whole group from 10.57 ± 1.92 to 7.00 ± 1.00 (*P *<* *0.05) nmol·L^−1^. The decrease for women was not significant, whereas corresponding decrease for men was from 13.61 ± 2.33 to 8.71 ± 1.17 nmol·L^−1^ (*P *<* *0.05). The difference in the reduction in testosterone between men and women was not statistically different. For the whole group IGF‐1 concentration showed a tendency for reduction from before to after the operation (*P* = 0.06). Women had 5.0 ± 0.5 and 4.4 ± 0.6 nmol·L^−1^, and men 4.8 ± 0.3 and 4.2 ± 0.3 nmol·L^−1^, respectively, without any differences between genders. Plasma levels of IL‐6 at rest were 12.9 ± 0.7 ng·L^−1^ with no differences between genders and with no significant increase following the exercise period.

### Muscle glycogen, CS, and HAD

These values from the muscle sample analysis are presented in Table 1. Muscle glycogen at rest was 269 ± 12 mmol·kg·dry·muscle^−1^ for the whole group (*n* = 21), which decreased to 181 ± 9 mmol·kg·dry·muscle^−1^ after the operation (*P *<* *0.05), with no difference in the decrease between women and men. For the whole group (*n* = 24) there were no major changes in the activity of CS or HAD after compared to before the eight days of exercise, also without any differences between the sexes.

**Table 1:** Levels of glycogen (mmol·kg^−1^·dry muscle) and activity of *β*‐hydroxyacyl‐Coenzyme A dehydrogenase and citrate synthase (mmol·min^−1^·kg^−1^·dry muscle), in muscle biopsies taken before and after the operation

	Pre	Post	Difference
Glycogen, mmol·kg^−1^ dry muscle			
All	261 ± 55 (181–431)	181 ± 89 (113–266)^a^	−88 (−116 to −60)
Women	286 ± 93 (198–431)	186 ± 33 (135–223)^a^	100 (−194 to −6)
Men	264 ± 40 (181–323)	179 ± 47 (103–226)^a^	−85 (−116 to −54)
HAD, mmol·min^−1^·kg^−1^ dry muscle			
All	41.1 ± 12.8 (22.1–63.3)	43.2 ± 12.5 (10.2–69.6)	2.1 (−9.8 to 5.6)
Women	37.3 ± 12.8 (19.5–52.3)	50.4 ± 11.5 (40.6–69.6)	13.1 (−6.9 to 34.8)
Men	42.9 ± 12.6 (19.7–63.3)	42.1 ± 8.8 (24.4–57.0)	−0.8 (−16.2 to 18.2)
*CS,* mmol·min^−1^·kg^−1^ dry muscle			
All	41.7 ± 9.7 (37.7–68.1)	45.3 ± 14.6 (15.1–93.5)	3.6 (−1.3 to 8.5)
Women	37.2 ± 6.2 (24.1–43.6)	43.8 ± 15.4 (15.1– 61.6)	6.7 (−21.6 to 8.2)
Men	43.6 ± 10.4 (31.7–68.1)	45.9 ± 14.7 (35.6–93.5)	2.3 (−2.5 to 7.1)

Difference refers to the change from Pre to Post. The values presented are means ± SE and range for 23 subjects (All), 17 subjects (Men), and 6 subjects (Women). In the case of glycogen, data from one male and one female subject are missing.

a *P *<* *0.05 versus Pre, evaluated by Student's paired *t*‐test.

### Protein content and phosphorylation

**Figure 1:**
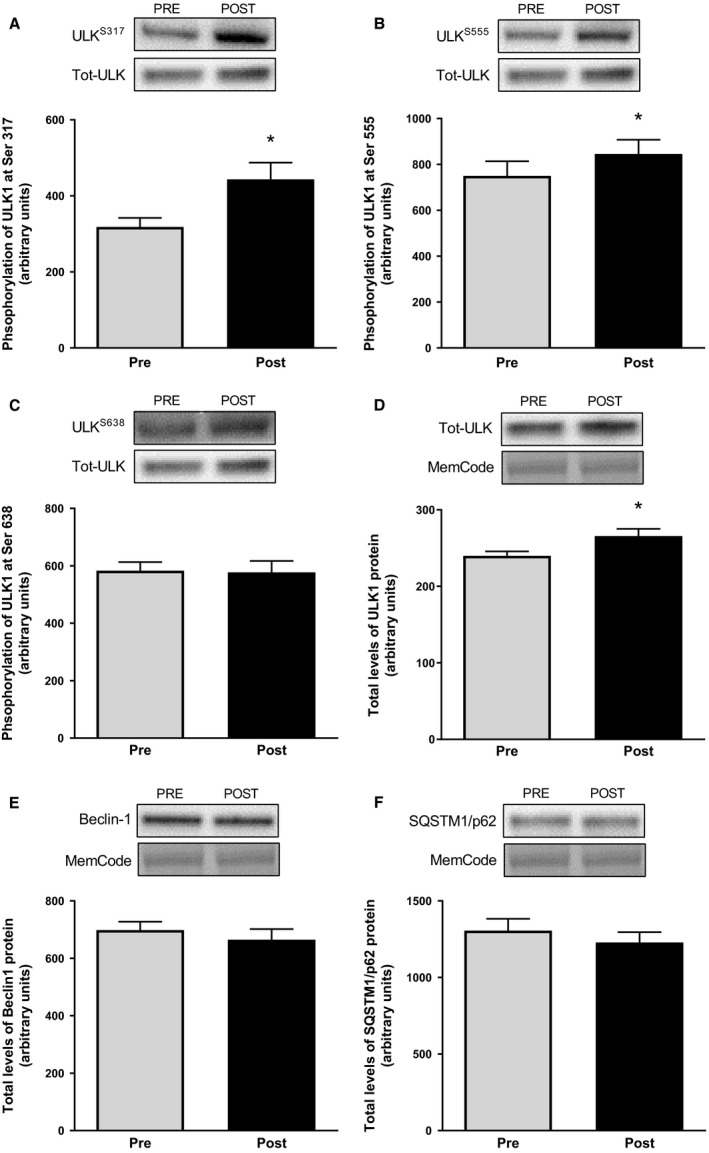
Phosphorylation of ULK1 at Ser^317^ (A), at Ser^555^ (B) and at Ser^638^ (C) as well as total protein levels of ULK1 (D), Beclin1 (E), and p62/SQSTM1 (F) in muscle biopsies taken before and after the operation. Representative blots are shown above each graph. The values presented are means ± SE for 13 subjects. *P <* *0.05 versus Pre, evaluated by Student's paired *t*‐test.

**Figure 2:**
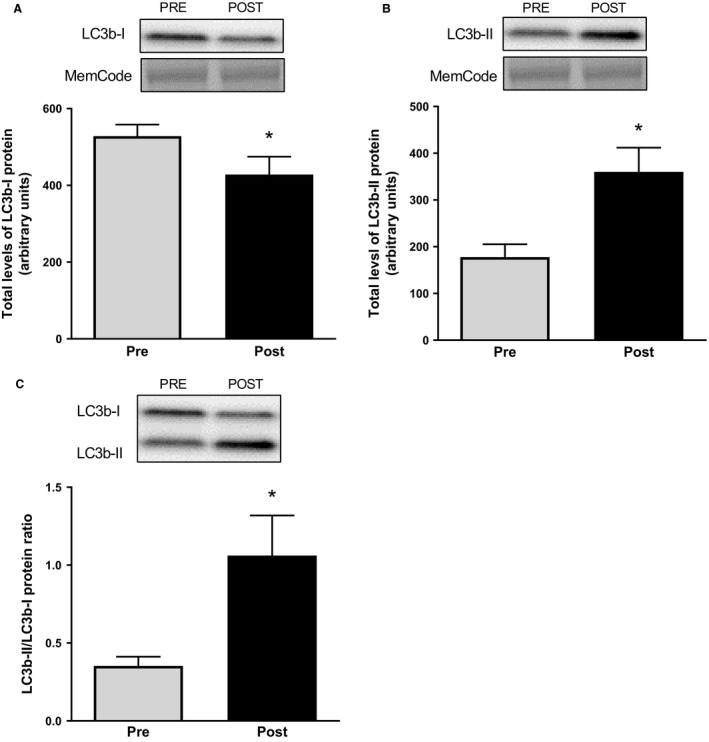
Total protein levels of LC3b‐I (A) and LC3b‐II (B) as well as LC3b‐II/ LC3b‐I protein ratio in muscle biopsies taken before and after the operation. Representative blots are shown above each graph. The values presented are means ± SE for 13 subjects. *P <* *0.05 versus Pre, evaluated by Students′ paired *t*‐test.

The phosphorylation of ULK1^Ser317^ and ULK1^Ser555^, both AMPK‐mediated, were increased after compared to before exercise with 39% and 13%, respectively (*P *<* *0.05, Fig 1A and B). This was not the case for ULK1^Ser638^ (Fig. 1C), which is mTORC1‐mediated and was unaltered following the exercise period. Total levels of ULK1 protein increased, slightly but significantly, by 11% after the operation (*P *<* *0.05, Fig 1D). Protein levels of Beclin1 and p62/SQSTM1 were unaltered after the exercise period as compared to before (Fig 1E and F). Protein levels of LC3b‐I decreased by 19% following the exercise period, whereas the lipidated form LC3b‐II increased by 104% in relation to preexercise values (*P *<* *0.05, Fig 2A and B). This resulted in a 3‐fold increase in LC3b‐II/LC3b‐I ratio following exercise (*P *<* *0.05, Fig 2C).

**Figure 3:**
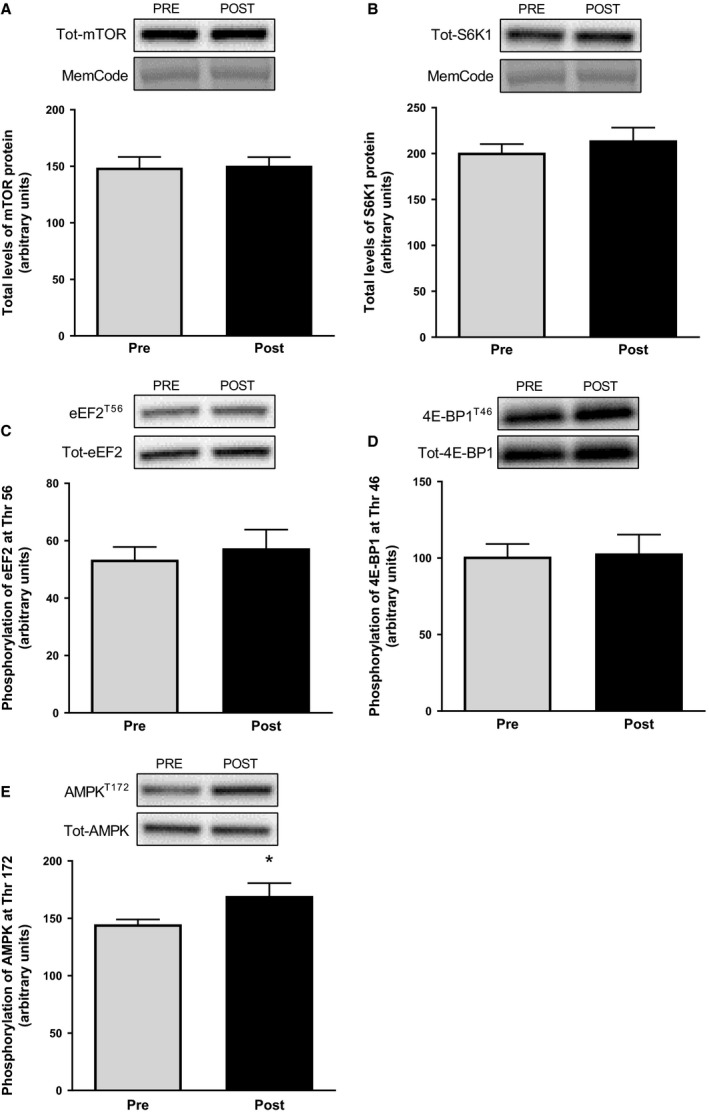
Total protein levels of mTOR (A) and S6K1 (B) as well as phosphorylation of eEF2 at Thr^56^ (C), 4E‐BP1 at Thr^46^ (D), and AMPK at Thr^172^ in muscle biopsies taken before and after the operation. Representative blots are shown above each graph. The values presented are means ± SE for 13 subjects. *P <* *0.05 versus Pre, evaluated by Student's paired *t*‐test.

The total level of mTOR (Fig 3A), S6K1 (Fig 3B), eEF2, and 4E‐BP1 protein did not change during the exercise period. This was also the case for the phosphorylation status of eEF2^Thr56^ and 4E‐BP1^Thr46^ (Fig 3C and D), indicating no suppression of mTORC1‐signaling. However, the phosphorylation of AMPK^Thr172^ increased by 17% from before to after the operation (*P *<* *0.05, Fig 3E), with the level of total AMPK protein being unaltered.

**Figure 4:**
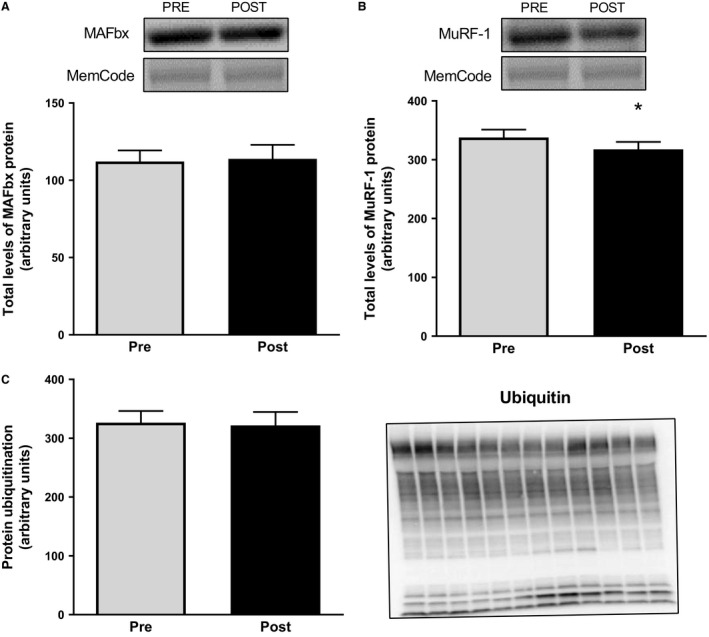
Total protein levels of MAFbx (A) and MuRF‐1 (B) as well as total protein ubiquitination (C) in muscle biopsies taken before and after the operation. Representative blots are shown above the graphs for MAFbx and MuRF‐1, and on the right of the graph for ubiquitination. The latter show lanes with Pre and Post samples alternating from left to right. The values presented are means ± SE for 13 subjects. *P <* *0.05 versus Pre, evaluated by Student's paired *t*‐test.

The protein content of MAFbx (Fig. 4A) did not change during the exercise period, whereas the protein content of MuRF‐1 decreased by 6% (*P *<* *0.05, Fig 4B), with no differences between men and women. The degree of protein ubiquitination did not change from before to after the exercise (Fig 4C) this was also the case for the level of free ubiquitin (data not shown).

### Proteasome activity

**Figure 5:**
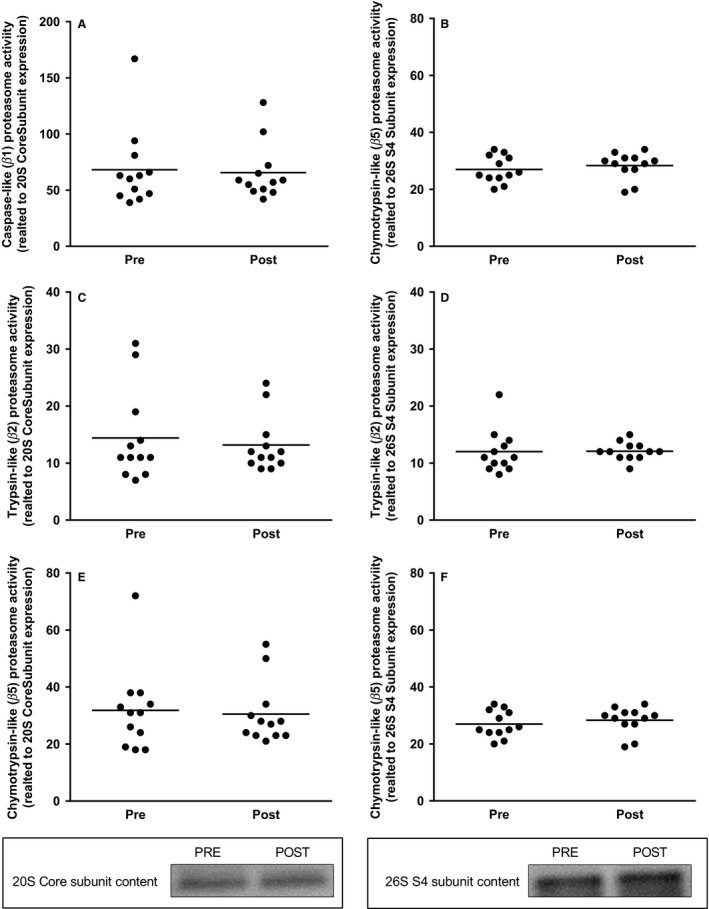
Caspase‐like (*β*1), Trypsin‐like (*β*2), and Chymotrypsin‐like (*β*5) proteasome activity related to 20S proteasome core subunit content (A, C, E) and related to 26S S4 subunit content (B, D, F), in muscle biopsies taken before and after the operation. Each symbol represents the activity for an individual subject. Representative blots for the proteasome content are shown below the graphs. The values presented are means ± SE for 12 subjects. *P <* *0.05 versus Pre, evaluated by Student's paired *t*‐test.

The chymotrypsin‐like (*β*5), trypsin‐like (*β*2), and caspase‐like (*β*1) activity of the proteasome did not change from before to after the exercise period, and there were no differences in activity between the sexes (9 men, 4 women). Normalizing the proteasome activity against the protein abundance of the 19S S4 subunit reduced the variation between subjects compared to normalization against 20S core subunit abundance, but did not alter the outcome (Fig. 5).

## Discussion

The aim of this study was to increase our knowledge of the molecular processes controlling skeletal muscle protein turnover in relation to an 8 day, field based, military training operation consisting of low‐ and high‐intensity exercise combined with a negative energy balance. The average energy expenditure was approximately 3 kcal·kg^−1^·h^−1^ during the whole operation, including periods of rest and sleep, resulting in a ~25% or ~1350 kcal per day energy deficit and *a* > 4% loss of body weight. The physically demanding military operation led to an induction of autophagy based on the increased AMPK‐mediated phosphorylations of ULK1, together with increased total protein levels of ULK1 as well as a increase in LC3b‐II/LC3b‐I ratio. In contrast to our hypothesis there were no increase in *β*1, *β*2, or *β*5 activity of the proteasome and the protein level of MuRF‐1 were slightly reduced. Moreover, protein levels of key components in the mTORC1 signaling pathway as well as phosphorylation of 4E‐BP1^Thr46^ and eEF2^Thr56^ remained unaltered, suggesting that signaling proteins involved in the regulation of protein synthesis were not reduced by the exercise period/nutrient deprivation.

We are fully aware of the problems to quantify both type and intensity of the physical activity as well as the total amount of energy ingested and the nutritional composition of the food provided to the participants under field conditions. However, the estimated energy expenditure in this study using heart rate recordings are well in line with energy expenditures quantified under similar military training operations using doubly labeled water (Hoyt et al. 2006; Margolis et al. 2013, 2014). As energy intakes were estimated based on rations provided to the soldiers they must be considered rough estimates as meals may have been omitted and in between meal snacks may have been added. However, there is no doubt that the energy deficit was significant and according to the estimates the women and the men should have lost approximately 1 and 2 kg of tissue mass, respectively. Adding reasonable fluid losses of about 2 L to that it fits well with the ~4% total body mass lost among the soldiers, and argues that the energy deficit estimates are quite reliable and not overestimated.

### Autophagy

The assessment of autophagy in human skeletal muscle is limited by the fact that autophagic flux assays are not available for in vivo human muscle samples. Nevertheless, quantification of LC3b‐I/II protein levels is considered a valid indicator of autophagosome synthesis and when combined with analysis of upstream signaling events and determination of p62/SQSTM1 protein levels as an indicator of autophagosome clearance an overview of autophagy can be formed (Klionsky et al. 2016b). Here, LCb3‐I levels decreased and levels of lipidated LC3b‐II increased, resulting in a threefold increase in LC3b‐II/I ratio, suggesting an increase in autophagosome formation. These findings are supported by the upstream activation of ULK1 as indicated by the increased phosphorylation at its Ser^317^ and Ser^555^ residues which promotes ULK1 activity (Egan et al. 2011; Kim et al. 2011b). We also report a significant increase in ULK1 total protein levels following the prolonged and exhausting exercise period. To our knowledge the latter is a novel finding following exercise in human skeletal muscle and suggests an increased capacity to stimulate autophagy. Formation of the autophagosome involves arrangement of the Beclin1/Vps34 complex (Eskelinen and Saftig 2009) where Beclin1 is shown to be required for autophagy (Liang et al. 1999). We found no change in levels of Beclin1 protein following the exercise period which is in line with previous work in humans showing increased autophagy after exercise (Jamart et al. 2012; Moller et al. 2015) arguing that exercise stimulated autophagy is not regulated by the protein levels of Beclin1 *per se*, but may of course be dependent on its binding capacity to Vps34.

While the ULK1 and LC3b data argue for increased autophagy, protein levels of p62/SQSTM1 did not change which suggests no increase in autophagosome clearance or a block in autophagic flux. Interestingly, previous studies utilizing conventional durations of exercise (≤2 h) all report reductions in LC3b‐II/I ratio along with activation of upstream signaling (Glynn et al. 2010; Fry et al. 2013; Moller et al. 2015; Schwalm et al. 2015; Fritzen et al. 2016) whereas Jamart et al. (2012) reported an increase in LC3b‐II/I ratio after a 24 h ultramarathon. Unfortunately the latter authors did not measure p62/SQSTM1 protein levels but together with our present data it could be argued that conventional exercise increase autophagic flux, but if exercise is severely prolonged there is reduction or block in flux, either as a protein sparing mechanism or due to some limitation in lysosomal clearance. It must also be noted that exercise can stimulate the mRNA expression of p62/SQSTM1 (Jamart et al. 2013; Schwalm et al. 2015). Accordingly, a reduction in p62/SQSTM1 protein levels due to autophagosome clearance could have been masked by an increased de novo synthesis of the protein. Although it was out of scope for this study a time course of the changes in LC3b‐II/I and p62/SQSTM1 would have formed a better descriptive picture of the changes in autophagy.

The ULK1 phosphorylation sites Ser^317^ and Ser^555^ are shown to be AMPK‐mediated (Egan et al. 2011; Kim et al. 2011b) which is in accordance with the observed increase in AMPK*α* ^Thr172^ phosphorylation in this study. The increase in AMPK^Thr172^ phosphorylation was somewhat unexpected as previous studies have reported no change following energy deficit, fasting, low‐intensity exercise or a combination of the latter two (Wojtaszewski et al. 2000; Vendelbo et al. 2012; Areta et al. 2014; Schwalm et al. 2015). It is, however, possible that increases in intracellular [Ca^2+^], maybe due to ER‐stress, could have stimulated AMPK phosphorylation and subsequent autophagy (Woods et al. 2005; Hoyer‐Hansen and Jaattela 2007).

Although we did not specifically asses mitophagy, the unaltered activities of both HAD and CS argues that the stimulation of autophagy has not had a significant impact on mitochondrial protein levels. Especially as the activity of CS has been shown to be a good marker of mitochondrial content (Larsen et al. 2012).

### mTORC1 signaling

ULK1 is also under control by mTORC1 which phosphorylates the Ser^638^ and Ser^758^ under amino acid rich conditions to reduce autophagy (Shang and Wang 2011). The phosphorylation of ULK1^Ser638^, 4E‐BP1^Thr46^, and eEF2^Thr56^ was unaltered after the exercise period suggesting that mTORC1 signaling remained at baseline and did not alter autophagy. Energy deficit has been shown to reduce mTORC1 signaling, but being rescued by resistance exercise and protein ingestion (Areta et al. 2014; Murphy et al. 2015). It is possible that the muscle work performed by the soldiers in this study prevented a potential energy deficit‐induced reduction in mTORC1 signaling. Total protein levels of mTOR, S6K1, 4E‐BP1, and eEF2 remained at resting levels after the operation which together with the phosphorylation data suggest that mTORC1 signaling and protein synthetic capacity is unchanged following 8 days of continuous exercise with a negative energy balance and moderate to high protein intake. Future studies utilizing methodology to measure fractional synthetic rate, for example, with deuterium oxide, are warranted to confirm the present findings under similar conditions.

### Ubiquitin–proteasome system

Several studies have reported exercise‐induced changes in the gene expression of MAFbx and MuRF‐1 (Yang et al. 2006; Louis et al. 2007; Mascher et al. 2008; Borgenvik et al. 2012b; Apro et al. 2015), but only a limited number of studies have assessed proteasome activity in relation to exercise. An increase in trypsin‐like (*β*2) proteasome activity has been reported following a 24 h ultramarathon (Jamart et al. 2012), whereas conversely, the same group reported a decrease in *β*5 activity after a 200‐km run (Kim et al. 2011a). In addition, no study has shown altered proteasome activity following exercise with short to moderate duration. Due to the strenuous nature of the operation we expected proteasome activity to increase but in light of the inconsistent previous findings our data are not surprising, and actually in accordance with the unaltered degree of protein ubiquitination as well as the stable levels of MAFbx protein. Even though protein levels of MuRF‐1 were reduced, the quite minor change (−6%) cannot be expected to have a major impact on proteasome activity, although it suggests a reduced capacity for proteasome breakdown and a protein sparing mechanism. Furthermore, Baehr et al. (2014) showed that during a period of plantaris muscle functional overload MuRF‐1 expression increased only in the early stage, whereas 26S proteasome activity increased as hypertrophy progressed. Accordingly our two time point data are intricate to evaluate. We might simply have missed alterations present in the early stages of the operation as reported by Jamart and colleagues (Jamart et al. 2012) after 24 h of running, which might have contributed to the presumed loss of lean tissue. Moreover as ingestion of food containing a moderate amount of protein has been shown to reduce proteasome activity in human skeletal muscle (Lunn et al. 2012; Carbone et al. 2013) it is conceivable that the 1.8–2.0 g of protein per kg body weight the soldiers ingested per day might have attenuated an exercise‐induced increase in proteasome activity.

### Metabolic and hormonal changes

As expected the consequences of the energy deficit were reduced body weight, increased plasma FFA and decreased plasma cholesterol concentration. In addition, muscle glycogen content was reduced to an average of 181 ± 9 mmol·kg·dry·muscle^−1^, which is similar to levels observed after ultraendurance events or glycogen depleting exercise protocols (Borgenvik et al. 2012ba; Psilander et al. 2013). However, preoperation levels were surprisingly low, in fact almost half the amount of muscle glycogen normally seen in well rested and fueled subjects of similar fitness (Psilander et al. 2013; Moberg et al. 2016). This indicates that the soldiers’ daily activities are of an intense nature and particularly that their nutritional intakes are poorly managed and operation preparation was inadequate.

The reduction in testosterone, specifically for the men, during prolonged exercise and energy deficit has been observed earlier (Marniemi et al. 1984; Viru et al. 1992; Berg et al. 2008). The postoperation concentration noted for the males is classified as likely physiologically insufficient and argues for a reduced capacity for protein synthesis, however, independent of mTORC1. Cortisol is known to be an important factor in promoting skeletal muscle atrophy, for example, by reducing muscle protein synthesis through inhibition of mTOR activity or by stimulating proteolysis through increasing atrogene expression (Schakman et al. 2013), which on the contrary can be downregulated by IGF‐1 (Stitt et al. 2004). Neither cortisol nor IGF‐1 was, however, changed in this study which is accordance with the unaltered protein levels and signaling through mTORC1 as well as the unaltered proteasome activity and levels of MAFbx. The slight reduction in MuRF‐1 protein is thus likely not mediated by changes in cortisol or IGF‐1 and suggests that another mechanism is involved.

Although we acknowledge that the employed submaximal exercise test is an insensitive measurement to accurately determine *V*O_2max_, the unchanged oxygen uptake per kg and min noted argues for a maintained aerobic effect with the soldiers. This is supported with the unaltered activities of both HAD and CS, as changes in oxidative enzymes are shown to be greater and precede significant changes in oxygen uptake (Coyle et al. 1984; Perry et al. 2010).

### Limitations

This field‐based study has great applied value but it comes with limitations. Monitored energy intake, energy expenditure assessed with doubly labeled water and proper assessment of body composition would have been of great value in the evaluation of the catabolic state. Furthermore, the assessment of protein synthesis using deuterium oxide would have been preferable, especially for relating the mTORC1‐signaling to a more robust outcome. As evaluation of autophagy via western blotting has its limitations further assessment of autophagosome location and content using immunohistochemistry would have added to the conclusion. Finally, a time course in changes of the molecular processes studied would have increased our understanding, but was not practically possible due to the risk of infections in damp and nonsterile environments following a muscle biopsy.

## Conclusion

The 8‐days of low‐intensity exercise with bouts of intense exercise and a concomitant energy deficit of about 25% resulted in an <4% reduction in body mass, loss of arm, and leg strength, very low levels of muscle glycogen and reduced levels of plasma testosterone, all in all representing a state of catabolism. With regard to the molecular regulation of muscle protein turnover, the operation resulted in stimulation of autophagy according to the increased phosphorylation of AMPK and ULK1 together with the increased LC3b‐II/I ratio. However, the latter also indicates a block in autophagic flux which together with the unchanged activities of all the *β*‐subunits of the proteasome and the slightly reduced levels of MuRF‐1 argues for a protein sparing effect toward the end of the operation. The unaltered protein levels and phosphorylation status of key components of the mTORC1 signaling pathway following the operation indicates a maintained capacity for protein synthesis. Future studies with multiple time point assessment of the molecular regulation and with direct measurement of protein synthetic rate are warranted.

## Conflict of Interest

No conflicts of interest, financial or otherwise, are declared by the authors.
